# Fluorescence spectroscopy for clinical transplantation liver grafts monitoring: possibilities offered by 408 nm excitation

**DOI:** 10.1590/acb370905

**Published:** 2022-12-12

**Authors:** José Dirceu Vollet-Filho, Juliana Ferreira-Strixino, Rodrigo Borges Correa, Vanderlei Salvador Bagnato, Orlando de Castro e Silva, Cristina Kurachi

**Affiliations:** 1 Ph.D. Universidade de São Paulo – Instituto de Física de São Carlos – Departamento de Física e Ciência dos Materiais – São Carlos (SP), Brazil.; 2 M.D., Ph.D. Universidade de São Paulo – Faculdade de Medicina de Ribeirão Preto – Departamento de Cirurgia e Anatomia – Ribeirão Preto (SP), Brazil.; 3 Ph.D. Universidade do Vale do Paraíba – Instituto de Pesquisa e Desenvolvimento – São José dos Campos (SP), Brazil.

**Keywords:** Transplantation, Liver, Fluorescence

## Abstract

**Purpose:**

Fluorescence spectroscopy techniques have been investigated aiming to reduce the invasiveness of methods for investigation of tissue. In transplantation procedures, it may offer the possibility of a complementary technique for the monitoring of liver grafts’ conditions prior to and during the transplantation procedure stages involving cold perfusion. The objective of this study was to evaluate fluorescence spectroscopy under violet light excitation (408 nm) for the monitoring of clinical hypothermic liver transplantation procedures.

**Methods:**

Organ grafts were monitored from before the removal of the donor’s body to 1 h after the implant into the receptor’s body. Fluorescence spectroscopy was assessed over five stages within these transplant stages.

**Results:**

The study provided evidence of a correlation between fluorescence information collected during liver grafts transplantation and the survival of patients.

**Conclusions:**

Fluorescence spectroscopy can become a tool to monitor transplantation grafts, providing objective information for the final decision of surgeons to use organs.

## Introduction

Fluorescence spectroscopy techniques have been investigated aiming to reduce the invasiveness of methods for investigation of tissue parameters and other relevant aspects. Fluorescence is based on exciting molecules with specific wavelengths and collecting the light emitted within a very short time interval (within nanoseconds from excitation). The spectrum of emitted light is directly dependent on the molecules present in the tissue, providing a tissue *optical fingerprint* 1.

Compared to ionizing radiation or magnetic resonance imaging approaches, fluorescence is a much more superficial interrogation technique, as excitation light penetrates only up to the superficial tissue layers. However, its appeal is its versatility when compared to traditional techniques, as it may provide an almost real-time assessment based on information from specific molecular profiles of tissues, with no actual tissue withdrawal, which is relevant for a conservative diagnostic technique. It also provides fast response since information is collected *in situ*. In contrast, techniques such as histopathology diagnosis cannot always be analyzed locally and may take from hours until days to provide a response due to the tissue preparation time for microscopy analysis.

The motivation for using fluorescence spectroscopy in transplantation procedures is the possibility to offer a complementary technique for the monitoring of liver grafts’ conditions prior to and during the transplantation procedure stages involving cold perfusion. Liver metabolism is mostly suppressed during these stages, by both exchanging blood for a preservation solution and low temperatures to which the graft is subjected during cold perfusion and back-table stages. The metabolic stress of blood reperfusion associated to eventual failures in the initial perfusion process may both contribute to general failure of the transplantation procedure, due to irredeemable damages to the organ graft leading patients to death. Consequently, the ability to observe molecular aspects in a potential real-time application becomes attractive as a monitoring tool.

Thus, the main objective of this study was to evaluate the ability to explore fluorescence spectroscopy under violet light excitation (408 nm) for the monitoring of clinical hypothermic liver transplantation procedure. This study aims to provide evidence of the correlation between fluorescence information collected during liver grafts transplantation and the survival status of patients.

## Methods

### Liver grafts

Fifteen transplanted liver grafts were investigated. Deceased donors provided the grafts for patients from the Liver Transplantation Unit of the General Hospital at the Medical School of Ribeirao Preto of the University of São Paulo (HC-FMRP-USP). Both the study and the transplantation procedures have been approved by the Internal Review Board from FMRP-USP (approval reference number 5295/2005) and performed under the Brazilian federal laws. The family of every patient included in this study was informed on the clinical study and authorized inclusion by signing an Informed Consent Form.

Grafts were obtained exclusively from deceased donors, after brain death was confirmed and informed by the Region 2 Office for Transplantation Organs Recovery and Distribution of the São Paulo State (CNCDO2). Inclusion and exclusion criteria for donors were those used for the transplantation protocol of liver transplantation of the Health Secretary from the São Paulo state (Brazil).

Once listed for surgery, regular follow-up was performed on those patients at the liver transplantation medical clinic of FMRP-USP General Hospital. Clinical and biochemical parameters were monitored to assess metabolic function alteration during the patient follow-up, in order to update a patient rank in the transplant waiting list.

### Fluorescence detection

The device used for fluorescence spectroscopy is a prototype assembled at the São Carlos Institute of Physics of the University of São Paulo (IFSC-USP) with excitation at 408 nm. The optical fiber probe delivers light from the laser to the target tissue (by perpendicular positioning onto tissue surface for light coupling), and simultaneously collects light emitted from tissue delivering it to the spectrometer. Relative intensity of fluorescence graphs were generated as a function of wavelength. A sterile, flexible Tegaderm film was placed between probe tip and liver surface to avoid cross-contamination and probe damaging.

### Procedure

Donors were directed to surgery for graft recovering. After the hepatectomy, the graft is positioned in an orthotopic position according to the piggyback technique^6^^–^ 8.

This study was conducted throughout the whole transplantation procedure. Patients underwent surgery between April 2006 and May 2009. Fluorescence assessment was performed by collecting five spectra from random spots on the surface of each of the medial right lobe, lateral right lobe and lateral left lobe of livers, for a total of 15 spectra per stage of collection.

There were five stages of collection, according to the main transplantation stages. The first stage collection was performed during recovering, being Stage 1 immediately after opening of the donor’s peritoneal cavity, when liver is completely functional in the donor’s system, and from which the natural endogenous fluorescence is obtained (named *autofluorescence,* AF). Next two collection stages were performed during cold perfusion, under hypoxic conditions: Stage 2 was performed immediately after establishment of *cold perfusion* (CP), and Stage 3 was performed by the end of *back-table* (BT) stage, immediately prior to receptor’s anastomoses. The last two collection stages were performed after warm reperfusion: Stage 4 was performed *5 min after blood warm reperfusion* (WR5), and Stage 5 was performed *60 min after reperfusion* (WR60).

Spectra were averaged per lobe portion in which they were collected, and an average of the five spectra was used to represent the spectrum for each lobe, at each stage. Fluorescence intensity at the portions of maximum emission of the spectra (the emission peaks at 442, 507, 556, 605, and 685 nm) were compared and correlated among them at every stage of collection and compared with information from biopsies collected at each of the five stages and with post-transplantation status of receptor patients (survivor or deceased).

### Optical analysis

Fluorescence spectra were processed using the software OriginPro (OriginLab, MA, USA). The average spectra obtained for each liver lobe was collected for each patient, for a total of three average spectra per stage, and fifteen average spectra for each procedure.

Every average spectrum was processed as follows: baseline correction was applied to reduce noise. Since liver fluorescence generated by 408 nm excitation produces several emission bands, the maximum intensity of the fluorescence spectrum at each of the emission peaks was obtained from the spectra, and these maxima were correlated among them on a two-by-two ratio. This data treatment allowed observing changes in the shape of the spectra over the procedure, which reflect physical and metabolic changes in tissue. Those changes can generate an increase or a decrease in the emission peaks intensity, as changes in molecules concentration modify absorption and emission of fluorescence, thus modifying the ratio between spectral bands’ height and width.

The survival status of receptors was considered during the first 30 full days after the transplantation procedure. Death within this period was considered as a failure on transplantation, otherwise considered successful (patient survival).

### Biochemical tests

Venous blood was collected at the beginning of recovering surgery and both 5 and 60 min after warm reperfusion, for the assessment of aspartate transaminase (AST), alanine transaminase (ALT), lactic dehydrogenase (DHL) and direct bilirubin (DBb) dosages, which were compared to fluorescence information. Blood serum ALT and AST aminotransferases, DBb and DHL from the beginning of recovering and from both 5 and 60 min after warm reperfusion were tested, and again from the first to the sixth postoperative day. ALT and AST aminotransferases and DHL were evaluated by the kinetic method at 340 nm, and the obtained values were expressed in U/L^9^^,^ 10; DBb was evaluated by colorimetric method at 525 nm, and values expressed in mg/dL^11^.

## Results

### Typical fluorescence spectra obtained at 408 nm excitation

Typical spectra observed for liver fluorescence emission at 408 nm excitation obtained from a patient whose transplantation procedure was successful with no adverse intercurrences for the patient’s clinical status post-procedure were obtained for reference, and can be found in the Supporting Information (please see the “Data Availability Statement” at the end of the manuscript).

### Spectral analysis

**Figure 1:**
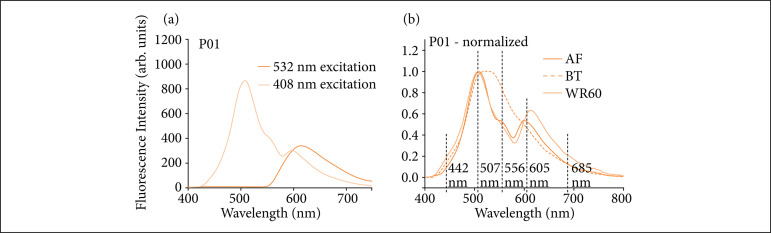
Comparison between fluorescence spectra at 532 and 408 nm excitation, with examples of spectra obtained for patient P01. **(a)** Note the only the band centered around 600 nm is observed for both spectra, whereas 408 nm excitation provides other spectral regions to be analyzed; normalized spectra, indicating the peak absorption wavelengths observed for different stages: endogenous fluorescence (AF) and back-table (BT) stages prior to organ graft implantation, and 60 min after anastomoses finalization (WR60); **(b)** The main decrease in fluorescence when compared to AF was about 556 nm (green spectral region with intense light absorption by blood constituents).

The emission bands most observed in all patients’ spectra throughout the transplantation procedures were centered at wavelengths 442, 507, 556, 605, and 685 nm. Figure 1 shows both the difference between spectra obtained by 408 and 532 nm excitation^6^ and the positions of those wavelengths.

### Patterns observed in surviving vs. nonsurviving patients

Different forms of analysis were performed, seeking patterns that would allow to adequately classifying any set of alterations observed in transplanted grafts in terms of the survival of the patient during the first 30 days after the procedure.

**Figure 2:**
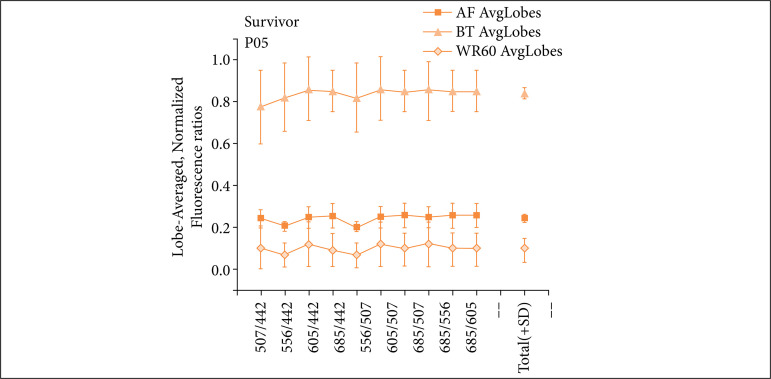
Ratio between emission maxima (RBEM) of fluorescence: typical surviving patient. The RBEM for wavelengths 442, 507, 556, 605 and 685 nm (patient P05; the graph represents typical results for surviving patients; others can be seen in the Supporting Information).

**Figure 3:**
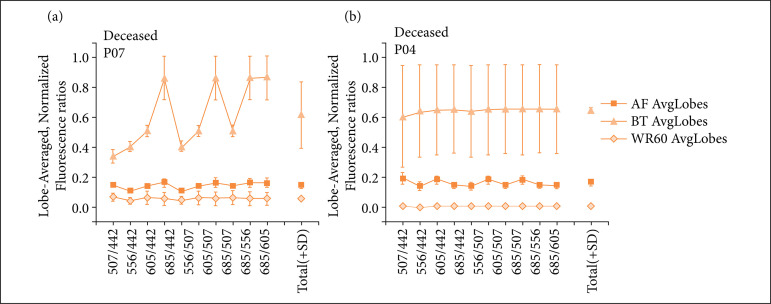
RBEM of fluorescence for wavelengths 442, 507, 556, 605 and 685 nm: examples of typical results for deceased patients.**(a)** patient P07 – large difference of the RBEM at BT stage regarding wavelengths; **(b)** patient P04 – large variance for all RBEM at BT stage regarding the points assessed (represented by standard deviation variance).

Figures 2 and 3 show ratios between wavelengths assessed for the main transplantation stages: AF, as this is the moment when liver tissue is considered under optimal conditions, still in the donor’s body; BT, because at this stage the preservation solution had time enough to completely accommodate, filling in the vascular system of the organ graft, which takes place not before 2–3 h of cold perfusion; and WR60, i.e., 1 h after finishing the main cycle of anastomoses, at which point the graft should be back to its regular functioning, after overcoming the metabolic and oxidative stress provoked by warm reperfusion. In these figures, error bars represent standard deviation of measurements among the three main liver lobes. Lines connecting dots are visual guides only. The rightmost dot in the graph represents the average (with standard deviation) among different RBEM for each transplantation stage presented, so that deviation represents divergence among ratios for the same stage. The “SURVIVOR” label means that the referred patient survived the first 30 days post-transplantation. The “DECEASED” label means that a patient has not survived the first 30 days post-transplantation.

Wavelengths of choice are related to the emission of the following substances in liver for 408 nm excitation^12^: 442 nm: collagen/elastin, bound reduced nicotinamide adenine dinucleotide phosphate (bound NAD(P)H); 507 nm: collagen/elastin, vitamin A; 556 nm: flavins; 605 nm: protoporphyrin IX (PpIX); and 685 nm: PpIX.

On the other hand, Fig. 3 shows that half of the patients deceased within 30 days of the transplantation presented some sort of relevant alteration on spectra, which can be appreciated by the RBEM values.

**Figure 4:**
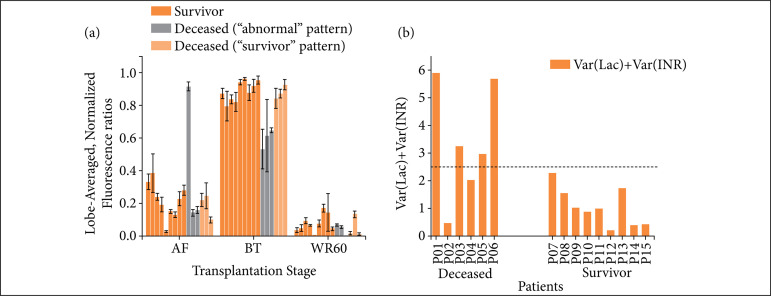
Average fluorescence ratios and relevant biochemical variations for surviving-versus-deceased patients. **(a)** Averages per patient of the RBEM of fluorescence, according to the transplantation stage; these averages show that three of the six cases of unsuccessful transplantation procedures show a deviant behavior of the RBEM, which indicates a distortion of the spectra during the process; **(b)** The joint variation of lactate and prothrombin per patient is shown. The relative variation indexes of lactate (Lac) associated with prothrombin (INR) were used for assessment. About 67% of deceased patients showed simultaneously high values of relative Lac and INR. When the relative variation indexes are summed up, these values reached over 2.5 for most deceased patients, in contrast to all surviving patients, suggesting a correlation between death and alterations undergone by the transplanted liver grafts.

Figure 4a shows the averages of RBEM for all the patients, according to the transplantation stage (AF, BT, and WR60).

### Correlation with biochemical data

Complementing fluorescence information, biochemical data collected from grafts/patients throughout the procedures were correlated between the biochemistry information and the survival status of patients 30 days after transplantation (Fig. 4b).

## Discussion

### Typical fluorescence spectra obtained at 408 nm excitation

In successful transplanted patients, the main change in fluorescence is the spectrum intensity. The difference between 408 nm spectra and those obtained at 532 nm^6^ excitation is promptly noticeable in Fig. 1. For the latter, a single, broad emission band, between 450 and 800 nm is found. In contrast, for 408-nm excitation, more energetic photons allow for electronic transitions among a broader range of electronic states, enabling emission of several energy bands—and thus broader and more varied emission spectra. The presence of several emission bands within a single spectrum is interesting because it allows one to compare specific portions of a spectrum within and among spectra, in order to seek repeating patterns for specific changes among the spectra of surviving (or nonsurviving) patients.

In addition to this, the change in the intensity of the spectra was observed, depending on the presence or the absence of blood. As hemoglobin is the major light absorber within the liver, when it is removed from the tissue there is a consequent increase in the fluorescence intensity, which takes place during cold perfusion stages—CP and BT. This increase is followed by an abrupt decrease in the fluorescence intensity due to the warm reperfusion, during stages WR5 and WR60. These findings are in agreement with what has been previously observed for the excitation at 532 nm by Castro-e-Silva *et al.* 6.

### Spectral analysis

The increase in the average fluorescence signal particularly at 550 nm is expected, as during the CP and BT stages blood has been completely removed from the graft tissue. Its absence implies the lack of important optical absorbers (such as hemoglobin) of the tissue, as can be seen in Fig. 1, resulting in an increase of the fluorescence signal as light is less absorbed. This enhancement in fluorescence is further increased by the decrease of temperature^13^. This finding corroborates a previous observation on spectra collected at 532 nm excitation for other emission bands—and it is worth noting that either bound or unbound forms of hemoglobin promote themselves optical absorption approximately between 530 and 600 nm^14^.

As can be seen in Fig. 1, the nature and variety of the intensity changes noticed at the collected spectra make difficult to identify patterns that allow one to correlate them to the survival status of transplanted patients. For further analysis, the ratio between the intensity of the wavelengths that indicate the peak of emission bands was observed.

These ratios were observed two-by-two in order to assess the variation of each emission peak according to each other. If the values for the RBEM are close, the spectral distortion for that region of the spectrum is negligible, which also means that the assessed tissues underwent little to none modification that might be detectable by its fluorescence emission at 408 nm excitation.

However, RBEM variation means that the amount of light emitted by the portion of the spectrum in question increased or decreased, which indicates that some level of metabolic alteration took place. This is attributed to changes in biochemical compounds or metabolic conditions of the tissue, as those changes can modify the availability of specific electronic transitions, which are responsible for the emission of fluorescence at specific wavelengths, or at any wavelengths at all.

### Patterns observed in surviving vs. nonsurviving patients

Similar RBEM values mean general maintenance of the average shape of the spectrum, which means that no relevant changes took place on tissue biochemistry. From the ratio among different peaks within a single patient, it is observed that all surviving patients produced fairly constant RBEM values, with small standard deviation. Similar values with small deviation mean that spectra for a single graft underwent little change on average. It is also noteworthy that RBEM values at WR60 stage become very similar to AF values, with ratios being similar among them. That behavior shows how fluorescence reflects the molecular profile of tissues with negligible variations among similar conditions, in agreement to what is observed for green light excitation as previously shown by Castro-e-Silva *et al.* 6.

Regarding the nonsurviving patients, the patients P04, P07 and P12 (Supporting Information) showed expressive deviation from the spectral behavior observed for succeeded patients, and a relative one for P08.

For P12, AF is the spectral portion with the largest overall RBEM values, which diverts from the pattern observed for surviving patients, as shown in Fig. 2.

For P07, there is an important increase in RBEM values at different ratios (and therefore different portions of the spectra), during back-table. At this point, RBEM average values were always the highest among those for a surviving patient, and never below 0.8; while for P07, RBEM values reach as low as 0.4 and 0.5, in contrast to other portions that do follow the pattern observed for survivors, clearly diverting from what is shown at Fig. 2.

For P04, average RBEM values on BT are far lower than average values observed for survivors, and error bars are much larger than for the other observations. These values indicate a divergence between changes occurring in different liver lobes (as lobes’ fluorescence was averaged to obtain standard deviation values). For the transplantation of patient P08, divergence was found for BT stage only, and exclusively for the RBEM 507/442, producing a low average value and larger standard deviation bars when compared to the average behavior for other RBEM of this organ graft.

Identifying these divergences on spectral behavior related to the demise of patients is an indication that unexpected and harmful alterations took place during the establishment of the grafts’ cold perfusion. The success of this stage is crucial for adequate preservation of the liver tissue, and thus any failure on full replacement of blood by the preservation solution may compromise the quality of tissue preservation and thus risk transplantation failure.

One cause of the alterations observed is the inefficient perfusion of the preservation solution, which is probably the main one. A subperfused organ graft—i.e., the insufficient replacement of blood by the cold preservation solution—will result in ineffective cooling of the graft and/or inability in maintaining acceptable levels of nutrients and quenchers of reactive species in order to keep portions of the tissues viable.

Figure 4a helps one to make a clearer idea of the sort of differences that can be found during transplantation. Part of the deceased patients shows a clear deviation from what was observed for all the surviving patients, particularly concerning the BT stage. More on the general observation made can be found on Supporting Information, where the analysis of RBEM for different stages of every patient was made available to readers.

In addition to the consequences for the general quality of the organ graft, failures in tissue preservation may produce harm during warm reperfusion, as the oxidative stress takes place, generated by the abrupt temperature increase, added to a sudden reestablishment of oxygen delivery and a fast, massive clearance of the preservation solution, taking away most of the oxidative stress quenchers that were available in tissue. Further, the temperature and pressure dynamics in vessels as fluids are exchanged may produce damage to the microvasculature. Inadequate preservation may potentialize this damage and therefore lead to tissue death, threatening the life of patients post-transplantation.

One of the aims of this study was to provide an approach that could indicate any failure in the procedure prior to implanting a graft. However, identifying parameters that would allow for *a priori* classification of expected procedure outcomes by fluorescence is not promptly achieved. Given the large variability of spectra from patient to patient, only BT and WR5 stages revealed a pattern on the fluorescence of surviving patients not reproduced for a deceased patient. Further discussion regarding this spectral analysis is provided as Supporting Information.

### Correlation with biochemical data

In spite of the volume of information provided, it was not possible to establish a direct correlation between the several aspects assessed by blood biochemistry and fluorescence. However, only the relative variation of lactate (Lac) and prothrombin (INR) before and after procedures showed a correlation with the survival status of patients^15^^,^ 16. Expressive and simultaneous variation in the relative levels of both parameters was evident for most deceased patients, in contrast to surviving patients. Since no significant information is obtained from this correlation, a more detailed discussion on these data is provided as supporting information.

### Final remarks on the obtained data and results

This study provided interesting comparisons between fluorescence spectroscopy and the survival status of patients receiving transplanted organ grafts, although a direct correlation was not observed for all cases.

The technique itself has inherent limitations as well, which challenge even more one’s ability to obtain relevant information from the collected data. Fluorescence spectroscopy is a noninvasive and very superficial technique—the average light penetration in liver tissue at 408 nm is a few hundred micrometers due to intense light scattering and absorption, which depends on the presence or absence of blood tissue as well. Nonetheless, limits are expected for the ability to infer tissue properties based of superficial assessment from a technique that aims to provide info of a bulky tissue—the smallest thickness of a human liver is usually about centimeters long, and an adult human liver is estimated to have 2 L in volume, with large variation^17^.

The results of this study show the feasibility of fluorescence spectroscopy as an auxiliary monitoring technique for transplantation procedures, given that there are still several possibilities to explore concerning the best use of the acquisition information.

## Conclusion

This study aimed to investigate the use of fluorescence spectra (408 nm excitation) for different stages of transplantation of liver grafts from deceased donors, under hypothermic ischemia, in order to seek correlations to associate fluorescence spectra to the survival status of patients subjected to the procedure. Results showed that there is a correlation between the fluorescence obtained and survival status. In spite of the correlation observed, the type of alteration found was insufficient to warrant an effective classification method for successful/unsuccessful liver transplantations based on the survival of patients. A correlation was observed between the relative levels of lactate and prothrombin of patients (prior and postprocedure) and the survival status of receptor patients as well, but we could not correlate them to fluorescence information.

Such findings lead the way for further investigation on the use of fluorescence spectroscopy as a tool to investigate and monitor transplantation procedures. Expanding the numbers of patients—perhaps associated with other monitoring techniques—may provide an effective method to increase the survival of patients and the number of available organs through assessing the preprocedure and the maintenance of the liver graft quality prior to implantation.
